# Role of major histocompatibility complex variation in graft-versus-host disease after hematopoietic cell transplantation

**DOI:** 10.12688/f1000research.10990.1

**Published:** 2017-05-03

**Authors:** Effie W. Petersdorf

**Affiliations:** 1 Fred Hutchinson Cancer Research Center, Seattle, WA, USA

**Keywords:** MHC, hematopoietic cell transplantation, permissible HLA mismatch, haplotype, HLA expression

## Abstract

Graft-versus-host disease (GVHD) remains a significant potentially life-threatening complication of allogeneic hematopoietic cell transplantation (HCT). Since the discovery of the human leukocyte antigen (HLA) system over 50 years ago, significant advances have clarified the nature of HLA variation between transplant recipients and donors as a chief etiology of GVHD. New information on coding and non-coding gene variation and GVHD risk provides clinicians with options to consider selected mismatched donors when matched donors are not available. These advances have increased the availability of unrelated donors for patients in need of a transplant and have lowered the overall morbidity and mortality of HCT.

## Introduction

Patients with life-threatening malignant and non-malignant blood disorders can be cured through hematopoietic cell transplantation (HCT). Although siblings who share identical parent human leukocyte antigen (HLA) haplotypes remain the preferred donor, the probability that any given patient has a matched sibling donor depends on family size. For patients without HLA-matched siblings, the development of registries of unrelated donors worldwide has greatly facilitated the identification of suitable unrelated donors for transplantation ^1, 2^. In the half century since the discovery of the HLA system ^3– 6^, major advances in our understanding of the HLA barrier have made possible the use of donors with selected HLA mismatches and have provided many patients with the opportunity for a life-saving transplant, particularly patients of non-Caucasian ancestry. A complete understanding of the HLA barrier in graft-versus-host disease (GVHD) after unrelated donor HCT requires an appreciation of the unique features of the classical HLA genes, non-HLA loci resident to the human MHC, and the constituents of extended MHC haplotypes. This review describes the current state of the art for transplantation from unrelated donors. Significant advances in the use of HLA-mismatched haploidentical related donors with post-transplantation cyclophosphamide to overcome the effects of HLA mismatching on the non-shared haplotype allow successful transplantation for patients without unrelated donors ^7^. The reader is referred to some outstanding comprehensive reviews in the current practice of haploidentical transplantation ^8, 9^.

## The major histocompatibility complex

The human major histocompatibility complex (MHC) encodes a series of genes on chromosome 6p, of which the best known are the classical class I and class II loci. A formal definition of the MHC was made possible with complete sequencing of a reference haplotype of approximately 3.8 megabases (Mb) ^10^. Today, over 269 loci are recognized ^11^ and include four major classes of variation: protein coding, non-coding RNAs, small nucleolar RNAs, and pseudogenes. The HLA region is characterized not only by very high gene density but also by extensive sequence variation, particularly of the classical HLA loci. MHC loci are well known for their high degree of association with over 100 diseases ^12^. Together with the fundamental role of HLA proteins in the transplantation barrier, the MHC region remains one of the best-studied regions of the human genome.

HLA alloantigens were first identified in 1952 by way of complement-dependent microcytotoxicity assays that were informative for anti-HLA antibodies from blood donors and multiparous females ^3– 6^. Serologic methods were the mainstay of tissue typing early in the history of the field but since have been supplanted by molecular methods. Today, next-generation sequencing (NGS) platforms designed to comprehensively characterize large segments of HLA genes and in some cases establish the cis-relationship between markers provide investigators with an unprecedented view of sequence diversity and the organization of variation on haplotypes ^13– 16^. Use of molecular tools for HLA genes has led to the recognition of over 3,830 HLA-A, 4,647 HLA-B, 3,382 HLA-C, 2,011 HLA-DRB1, 1,054 HLA-DQB1, and 740 HLA-DPB1 alleles, just to name the six classical loci currently considered in the selection of HCT donors ^17^. The unique HLA allotypes are distinguished by substitutions at key residues that define the peptide-binding region (PBR) of the molecule. The nature of PBR substitutions reflects the antigen-presentation role of HLA molecules in host defense. In transplantation, the extraordinary degree of variation can present a major roadblock to the identification of donors for transplantation, as described below.

## Graft-versus-host disease after unrelated donor hematopoietic cell transplantation: the role for human leukocyte antigen alloantigens

GVHD is the second most prevalent cause of mortality after unrelated donor HCT ^18^. HLA mismatching remains one of the strongest risk factors for risk of acute and chronic GVHD and therefore upfront efforts to identify matched donors have been the mainstay of pre-transplant donor evaluation ^2, 19– 21^. The probability of finding HLA-matched donors depends on the race of the patient and on the composition of donor registries ^22^. Although international registries have high representation of donors of European Caucasian ancestry, this does not guarantee that Caucasian patients will always have matched donors. In general, the probability of finding HLA-A, -B, -C, and -DRB1 (HLA “8/8”)-matched donors is related to the parental alleles and haplotypes and their frequencies in donor registries ^23, 24^. An analysis by the National Marrow Donor Program (NMDP) of the Be The Match Registry demonstrated that the rate of matching all 10 alleles at HLA-A, -C, -B, -DRB1, and -DQB1 loci was lower for African-, Hispanic-, and Asian-Americans (<50%) than for Caucasian-Americans ^25^. To estimate the likelihood that a less-than-perfect match could be identified for any given patient, a follow-up analysis by the NMDP focused on the probability of finding HLA 7/8-matched donors (one mismatch at HLA-A, -C, -B, or -DRB1). Compared to Caucasian-Americans with 98% likelihood, Hispanic-, Asian-, and African-American patients had 86%, 88%, and 82% likelihoods, respectively ^26^. When matching considered all five loci, the probability of identifying a donor with a single mismatch (HLA 9/10) was 94%, 72%, 74%, and 61%, respectively. These data suggest that when an HLA 7/8- or 10/10-matched unrelated donor is not available, it is feasible to identify donors with one HLA mismatch.

Substantial data from transplant centers and transplant registries confirm the importance of complete and precise donor HLA matching to lower the risks of GVHD ^2, 19, 20, 27^; however, data concerning the acceptability of limited HLA mismatching when matched donors are not available are still coming into focus. A major impetus for understanding which mismatches can be used safely (i.e. are not associated with significantly increased risk of GVHD) and which mismatches should be avoided is the premise that relaxing donor selection criteria will substantially increase the odds of identifying donors for all patients. In fact, for patients of African-American background, over 80% will have donors mismatched for one HLA-A, -B, -C, or -DRB1 allotype ^26^.

To better understand the genetic basis for patient-donor mismatches that increase GVHD risk (and which should be avoided if possible), investigation has focused on the polymorphic exons that define the PBR of class I and II allotypes. For HLA-A, -B, and -C, this includes characterization of exons 2, 3, and 4, ** which encode the α1, α2, and α3 extracellular domains, respectively; whereas the α1 and α2 domains fold to form the PBR between two α helices on a β-pleated sheet platform, the membrane-proximal α3 domain encodes the main binding site for CD8 and contact residues for β2 microglobulin ^28, 29^. For class II HLA-DR and -DQ allotypes, definition of the transplant recipient and donor includes, at a minimum, the polymorphic exon 2, which encodes the residues that define the PBR.

## Qualitative and quantitative factors of human leukocyte antigen disparity in graft-versus-host disease risk

A role for donor-recipient mismatching at the classical HLA-A, -B, -C, -DRB1, and -DQB1 loci in GVHD risk has been amply demonstrated ^2, 19, 20, 27^. Investigation into the clinical significance of the sixth and last classical HLA gene, *HLA-DP*, required additional laboratory tools because traditional typing of *HLA-DP* gene products was performed through a secondary primed lymphocyte reaction which did not distinguish between allelic variants. Through a variety of different approaches, HLA-DP has been confirmed as a classical MHC locus in HCT ^30– 33^.

Early clinical experience demonstrated the high-risk nature of additive effects of multi-locus mismatching, which led to the practice of limiting the total number of HLA mismatches between the transplant donor and recipient ^34^. The importance of HLA mismatch dose was further illustrated with the HLA-C locus ^35^. Historically, donor matching included consideration for HLA-A, -B, and -DRB1; when retrospective analysis of HLA-C was feasible with molecular tools, many HLA “6/6”-matched transplants were retrospectively identified to have one or two HLA-C mismatches. The risk of graft failure increased with increasing number of HLA-C mismatches ^35^. Similar synergistic effects have been shown for HLA-DPB1 ^30^ and recently for the HLA-DRB1/-DRB3/-DRB4/-DRB5 loci ^36^. Updated analyses by the Japan Marrow Donor Program (JMDP) ^20^ and the Center for International Blood and Marrow Transplant Research (CIBMTR) ^2^ both confirm the deleterious nature of multi-locus HLA mismatching on GVHD and mortality. For this reason, limiting the total number of donor HLA mismatches to one will help to lower the risk of GVHD and mortality after HCT ^21^.

When matched donors are not available, research suggests that the judicious selection of donors with selected HLA mismatches may provide patients with a curative transplant without substantially increasing transplant-related mortality. These research studies have identified selected HLA mismatch combinations that differ at specific amino acid residues which define the class I PBR ^37, 38^. The JMDP experience has identified patient-donor mismatching at four key class I residues as risk factors for acute GVHD: Tyr9Ser, Tyr99Phe, Leu116Ser, and Arg156Leu were each associated with a significantly increased risk of acute GVHD among Japanese patients ^39^. Of these positions, mismatching at residue 116 was associated with increased mortality in a large retrospective analysis by the CIBMTR, providing independent validation of the importance of this epitope ^40^. At the HLA-DPB1 locus, *in vitro* cytotoxicity assays have been developed to evaluate the immunogenicity of amino acid residue mismatching ^41– 43^. Patient-donor mismatching for amino acid residues that define the hypervariable regions of DPβ is associated with GVHD risk and can be used to define combinations of patient-donor HLA-DPB1 mismatches that are associated with higher risks (“HLA-DPB1 non-permissive mismatches”) and those associated with GVHD rates not dissimilar to those observed among HLA-DPB1-matched transplants (“HLA-DPB1 permissive mismatches”) ^44, 45^. A clinically useful tool for evaluating patient-donor HLA-DPB1 mismatches has been developed with the aid of mutational studies ^46^. In an independent study, recipient residues encoded by the DPA1*01:03–DPB1*04:01 haplotype that define the HLA-DP peptide-binding pocket have been shown to correlate with sclerotic GVHD ^47^. These studies collectively support the hypothesis that motifs of the HLA-DP PBR are involved in GVH recognition.

Differences between the transplant donor’s and recipient’s HLA molecules at key amino acid residues within the PBR stimulate robust GVH alloresponses. In addition to such qualitative measures of GVH allorecognition are quantitative measures in which donor recognition of patient MHC differences is influenced by the amount of HLA protein expressed by host cells and tissues. Recently, demonstration that the level of expression of HLA-C and HLA-DP allotypes in patients with HIV and hepatitis B infection influence the course of these infections highlights the need for more complete information on the extent and nature of non-coding region variation within the HLA system ^48, 49^. Both the HIV-AIDs and hepatitis B models suggest that higher HLA expression promotes more effective presentation of virally associated minor antigens and enhanced host clearing of infection. Alternatively, lower HLA-C expression is protective in Crohn’s disease, suggesting a key role for HLA-C in the presentation of immunogenic antigens that participate in autoimmunity ^48^.

In HCT, the level of expression of HLA-C and HLA-DP mismatches in the patient is associated with risks of GVHD and mortality. HLA-C expression is allotype specific and follows a continuous spectrum of mean florescence intensity values, with HLA-C*03 and HLA-C*07 expressed at low levels compared to HLA-C*14, which is expressed at very high levels ^48^. Among transplants mismatched for one HLA-C antigen, as the level of the patient’s mismatched HLA-C allotype increases, the risk of GVHD increases ^50^. HLA-DP expression is influenced by the rs9277534 variant that resides in the gene’s 3’ untranslated region (UTR), where possession of the A allele is associated with low HLA-DP expression and G with high expression ^49^. Among transplants mismatched for one HLA-DP antigen, patients with low-expression HLA-DP mismatches had lower risk of GVHD compared to patients with high-expression HLA-DP mismatches ^51^.

The same loci that increase GVHD risk are also associated with lowered probability of relapse after transplantation. Known as the graft-versus-leukemia effect (GVLE) ^52– 55^, mismatching at HLA-C and HLA-DPB1 are most strongly associated with GVLE compared to mismatching at other classical HLA loci ^20^. The potent immunogenicity of HLA-DPB1 has recently been exploited in an *in vitro* model in which CD45RA-selected CD4 cytotoxic lymphocytes (CTLs) were stimulated with autologous dendritic cells that expressed HLA-DPB1 mismatched alleles on mRNA transfection ^56^. AML blasts that expressed the corresponding HLA-DPB1 alleles were directly lysed by the CTLs. These novel data provide a platform for the development of AML-reactive CTLs that use HLA-DPB1 as a potent target to mediate GVL responses.

## The role for major histocompatibility complex resident non-human leukocyte antigen loci in graft-versus-host disease

### Candidate gene studies

Genetic variation within the MHC is inherited *en bloc* in classical Mendelian fashion as a haplotype of markers on the same chromosomal strand. With current donor-matching criteria, less than 5% of the total MHC resident gene content is evaluated, and this opens up the potential for novel undetected variation as a cause of GVHD after HLA-matched transplantation. Together with HLA alleles, non-HLA loci in the MHC travel together, and their biological effects may synergize with those stemming from donor-recipient HLA mismatches. There has been strong interest in exploring non-HLA loci as a source of variation responsible for GVHD, particularly in the setting of HLA-matched transplantation ^57, 58^. Studies have approached the search for novel transplantation determinants through candidate gene approaches or fine mapping with the aid of single nucleotide polymorphisms (SNPs) that define blocks of tightly linked markers (tagSNPs). In the case of unrelated donors and patients, identity for HLA alleles does not guarantee identity for other MHC loci, particularly if the patient’s and donor’s haplotypes are different ^59^.

A growing number of studies have taken a candidate gene approach to explore the clinical significance of the non-classical class I loci including *HLA-E*, *HLA-G*, and MHC class I related chain A ( *MICA*). Each of these genes is polymorphic and each has unique features that make them interesting candidates for transplantation determinants. A total of 25 HLA-E alleles that give rise to 18 proteins have been recognized; however, two alleles, E*01:01 and E*01:03, are the most frequently observed in most populations studied thus far ^17^. Given its participation in both the innate and the adaptive immune pathways, *HLA-E* has been an attractive candidate gene with the potential to influence the risk of GVHD after transplantation; however, the evidence to date is heterogeneous ^60– 64^. In HLA-matched sibling transplantation, homozygosity for HLA-E*01:03 was protective for GVHD and associated with improved survival ^60, 62^. The presence of E*01:03 in transplant donors, however, was associated with higher grades (II–IV) of acute GVHD ^61^. Two additional studies did not find an association of HLA-E with clinical outcome ^63, 64^.

*HLA-G* is a non-classical class I gene best studied for its role in tolerance at the maternal-fetal placental interface ^65, 66^. *HLA-G* encodes a total of 53 allelic variants giving rise to 18 proteins ^17^. However, the most intriguing characteristic of this non-classical gene is its ability to form soluble as well as membrane-bound protein as a result of alternative splicing. A total of four membrane-bound and three soluble isoforms differ with respect to their size, structure, and ability to bind β2 microglobulin ^67, 68^. The transcriptional regulation of *HLA-G* is complex and includes, but is not limited to, genetic variation within the 5’ and the 3’ UTRs ^69– 71^. The 3’ UTR is particularly interesting, as it is characterized by a 14 basepair (bp) insertion/deletion and by haplotypes of SNPs, of which rs1063320 has been the subject of intense investigation as a basis for *HLA-G* expression and disease association ^71– 74^. The 14 bp insertion results in the removal of 92 bases from exon 8 and is correlated with lower expression of *HLA-G* transcript ^75, 76^. Its participation in both T and natural killer (NK) cell-mediated immune pathways ^68, 77– 80^ has prompted investigation into a role for *HLA-G* gene products in cancer, autoimmunity, and transplantation ^74, 79, 81^.

In HCT, homozygosity for the 14 bp deletion correlated with higher risk of acute GVHD compared to homozygosity for the 14 bp insertion ^82^. In an independent study, however, the 14 bp insertion was found to be a risk factor for acute GVHD ^83^. No correlation of the 14 bp insertion was found for acute GVHD, but an association with lower overall survival and disease-free survival was observed ^84^. Still, other investigations have not found associations among 3’ UTR haplotypes, the 14 bp insertion/deletion, and clinical outcome after HCT ^85, 86^.

Although it has classically been considered that HLA-G expression is restricted to the maternal-fetal interface, the thymus, and the cornea, several recent studies have found increased levels of HLA-G in the plasma ^87– 89^ and in GVHD target organs in patients receiving allogeneic HCTs ^87^. In the first 30 days after HCT, levels of the soluble G5, G6 and G7 proteins were significantly higher compared to pre-transplant levels; higher levels of soluble HLA-G proteins were found in patients without GVHD compared to lower levels in patients who developed grades II–IV acute GVHD ^89^. Very intriguing data on the recovery of CD14 ^+^ HLA-G ^+^ cells in the plasma of healthy and transplant patients and the ability to antagonize the suppressive function of these cells through HLA-G blockade provide new information on the contribution of HLA-G-expressing monocytes in the immune response ^87^. Transplant recipients were found to have a higher frequency of HLA-G ^+^ CD8 ^+^ T cells after transplantation. Furthermore, neo-expression of HLA-G in the epidermis of patients with clinical GVHD, and a direct correlation of HLA-G expression with the severity of skin GVHD, suggests that up-regulation of HLA-G is involved in the etiology or clinical manifestation of this disease. In an independent study of patients undergoing HCT for hematologic malignancies, high levels of soluble HLA-G proteins within the first month after HCT could be recovered in patients who did not develop acute GVHD; the level of soluble HLA-G proteins correlated with the frequency of T regulatory cells with the CD4 ^+^ CD25 ^+^ CD152 ^+^ phenotype in transplant recipients ^88^.

In addition to HLA-E and -G, the *MICA* gene has been an attractive candidate to explain GVHD risks in related and unrelated donor transplantation. A total of 106 unique alleles giving rise to 82 proteins are recognized ^17^. Although MICA shows sequence homology with HLA-A, -B, and -C, it lacks association with β2-microglobulin and does not bind or present peptides. MICA is expressed on the epithelium of the gastrointestinal tract (hence the interest in GVHD) and is induced by cellular stress ^90^. The MICA CD94/NKG2D activating receptor is expressed on most ϒδ T cells, αβ T cells, and NK cells ^91^. Engagement of MICA with NKG2D leads to NK-mediated killing of target cells and CD8-positive ϒδ T cell-mediated activation of CTLs ^92– 96^. It is no surprise then that MICA-NKG2D has been implicated in host resistance and susceptibility to infection ^97^, tumor surveillance ^98– 100^, and autoimmunity ^101– 103^. Particular attention has been paid to the rs1051792 variation that gives rise to methionine or valine at residue 129 of the MICA α domain; importantly, this position is associated with lower (valine) or higher (methionine) binding affinity to NKG2D ^104^ and impacts the strength of NKG2D signaling and co-stimulation of CD8+ T cells ^95, 97^.

Early investigation into the HLA-B–HLA-C region of class I uncovered donor-recipient mismatching for a series of markers, which were identified as MICA and MICB, and their potential relevance in HCT outcomes ^105^. Subsequently, a series of studies have shed light on the importance of donor-recipient MICA mismatching and the Met129Val polymorphism in clinical outcome after related and unrelated donor transplantation ^96, 106– 110^. The extensive linkage disequilibrium across the MHC favors MICA matching among transplant pairs who are HLA-A, -B, -C, -DRB1, and -DQB1-matched. The clinical significance of MICA mismatching on GVHD risk has been heterogeneous and is likely complicated by a low mismatch rate and population differences. Higher risk of acute GVHD, in particular GVHD of the gastrointestinal tract, has been observed with donor-recipient MICA mismatching in some ^106, 109^ but not in other studies ^107^. Recently, a large retrospective analysis of unrelated donor-matched and -mismatched transplants performed in Germany demonstrated higher risks of mortality, lower disease-free survival, and higher rates of acute GVHD with donor-recipient mismatching for Val129Met ^108^. The presence of 129Met in recipients correlated with better overall survival and lower risk of death due to GVHD; however, homozygous 129Met/Met patients had increased risks ^96^. A retrospective study of MICA performed by the CIBMTR found no association of MICA mismatching nor Val129Met on clinical outcome ^110^. The reasons for these heterogeneous results are unclear but may be the result of multifactorial genetic and environmental factors.

### Single nucleotide polymorphism mapping

The use of SNPs for fine mapping novel transplantation determinants within the MHC is founded on the concept that tightly linked markers serve as proxies for one another. The use of tagSNPs makes no *a priori* assumptions for the likely disease-causing genes or their associated pathways; for a region that contains the most immune-related genes anywhere in the genome, tagSNPs provide an efficient and robust way to locate novel transplantation determinants. The elucidation of conserved extended HLA haplotypes provided the much-needed reference sequence(s) for designing SNP arrays for disease mapping ^111– 113^. The availability of NGS methods has permitted the completion of in-depth interrogation of extended haplotypes that are homozygous for the classical HLA alleles, providing unprecedented annotation of coding and non-coding regions of the MHC ^114^. When the classical HLA-A1, -B8, -DR3 and HLA-A2, -B, -DR15 haplotypes are aligned, extreme levels of sequence conservation over 3 Mb in length are evident ^111^. Further appreciation of the nature and organization of genetic variation on extended HLA haplotypes is demonstrated in the analysis of common haplotypes in ethnically diverse populations. In Japan, for example, unrelated individuals show remarkable sequence conservation for the three most common HLA haplotypes, spanning 3.3 Mb in length ^115^.

Demonstration that haplotype-based approaches can facilitate the identification of novel transplantation determinants was shown in a study of HLA-matched unrelated donor-recipient pairs using a long-range phasing method ^116^. The physical linkage of HLA-A, HLA-B, and HLA-DR on the same chromosomal strand was performed to identify matched pairs with the same HLA-A, -B, and -DR haplotypes and matched pairs with different haplotypes. HLA-matched pairs with different haplotypes had a significantly increased risk of grade III–IV acute GVHD, lower relapse, and similar overall survival ^59^. These observations suggest that the HLA haplotype can be used as a surrogate marker for GVHD risk and for the identification of specific risk loci. To this end, SNP arrays have been used to query the MHC in matched and mismatched unrelated donor transplants. In HLA-matched transplantation, two SNPs were validated as determinants of survival and acute GVHD ^117^ and a similar strategy is informative for single-locus mismatched unrelated donor transplants ^118^. Collectively, the data thus far point to the presence of MHC resident variation that may confer risks alongside those stemming from HLA mismatching between the donor and the recipient. This information will enhance our understanding of the MHC as a critical region of the genome in transplantation biology and provide potential novel approaches for donor selection and for targeted immunotherapy.

## The role for non-major histocompatibility complex loci

Differences between the transplant patient’s and donor’s HLA class I and II gene products serve as potent antigens that stimulate GVH alloresponses, as described above. HLA class I genes also serve as the cognate ligands for NK cell receptors ^119^. Furthermore, differences in the peptides presented by the patient’s HLA class I and II molecules which are recognized by donor T cells as minor histocompatibility antigens represent an important source of variation that contributes to GVHD ^120^. For these reasons, the HLA system is a dynamic interface between the innate and adaptive immune systems, each of which has biological implications in HCT.

### Human leukocyte antigen ligands for natural killer cells

NK cells do not directly cause GVHD after HLA-matched or HLA-mismatched transplantation. Unlike T cells, which recognize recipient major HLA and minor histocompatibility differences in host leukemia and normal tissues, NK cells recognize target cells that lack class I ligands (missing self; missing ligand), a situation that may arise with viral infection or malignant transformation. Inhibitory KIR receptors interact with their cognate ligands during NK development, which induces tolerance to target cells ^121^. Target cells that are virally infected or are transformed by malignancy may lose their self-class I ligands; these target cells are recognized by licensed NK cells, leading to target cytotoxicity. In HLA-matched transplantation, patients who lack KIR ligands (missing ligand) experience lower risk of relapse and improved overall survival ^122^, consistent with cytotoxicity of host residual leukemia by unlicensed NK cells. In HLA-mismatched transplantation, wherein the patient lacks KIR ligands that are present in the donor, licensed NK cells can mediate cytotoxicity against the KIR ligand mismatch ^123^. The reduction in relapse in both scenarios is not accompanied by increased GVHD. These unique properties of NK cells are the basis for the development of NK cells in adoptive immunotherapy ^124^.

Inhibitory *KIR* genes demonstrate a range of allelic variation and the alleles may have higher or lower degrees of inhibition that add to the diversity of individual immune responses. One example illustrates that features of both the receptor, KIR3DL1, and the ligand, HLA-Bw4, can be associated with different risks of relapse and mortality depending on the strength of inhibition (high or low) and the specific residue 80 sequence polymorphism in Bw4-positive cells ^125^. An added layer of polymorphism in the KIR genetic system is the organization of genes into two major haplotype groups defined by the number and nature of genes with inhibitory and activating potential ^126^; whereas “A” haplotypes encode more inhibitory than activating genes, “B” haplotypes tend to encode more activating genes than do “A” haplotypes. Haplotype-based analyses of KIR demonstrate the importance of the number and nature of inhibitory and activating genes on A and B haplotypes on transplant outcomes. The presence of at least one B haplotype is associated with improved survival compared to lack of any B haplotype (i.e. only A haplotypes). These data support a role for activating KIRs in transplantation ^127^. Among the activating genes that have been studied thus far, the KIR2DS2 receptor and its HLA-C ligands expressing Asn77 and Lys80 (“C2” ligands) are capable of mediating strong anti-leukemic potential ^128^. The unique features of activating and inhibitory receptors together with their ligands provide avenues for lowering risk of relapse without an increase of GVHD through KIR-based algorithms applied to donor selection ^129^.

### Minor histocompatibility antigens

CD8 ^+^ and CD4 ^+^ T cells play a role in antigen-mediated recognition leading to GVHD after allogeneic transplantation. By definition, minor histocompatibility antigens are non-self peptides in which one or several polymorphisms within the homologous proteins between a transplant donor and patient may lead to altered binding of peptide to HLA and/or recognition of the HLA-peptide complex by T cells ^130, 131^. Due to the diversity of minor histocompatibility antigens that can be presented by HLA, donor T cell-mediated allorecognition of disparities in minor histocompatibility antigens presented by patient target cells may induce GVHD in both HLA-matched and HLA-mismatched transplantation from both related and unrelated donors.

The millions of nucleotide sequence variants within the human genome provide a rich source for potential minor histocompatibility antigens ^132– 134^. Variation within autosomal genes and genes of the Y chromosome contribute minor antigens of importance to transplantation ^135^. One of the best-studied minor histocompatibility antigens is the *HMHA1* gene-derived “HA-1” nonamer peptide presented by HLA-A*02:01 allotypes; for this minor antigen, both HLA and T cell receptor binding features contribute to the immunogenicity of HA-1 ^136, 137^.

Since SNPs represent the most common form of genetic variation accounting for the generation of minor histocompatibility antigens, the availability of SNP arrays to query whole genomes has yielded new insight into the loci that contribute to the pool of clinically relevant minor antigens ^33, 47, 138^. In a Japanese study of HLA 10/10-matched unrelated donor transplants, autosomal SNPs have been identified that significantly increase the risk of GVHD. The identification of the true causative genes awaits further investigation in independent cohorts.

To predict the extent to which patient mismatching for minor histocompatibility antigens can contribute to GVHD risk, a single-center GWAS analysis compared the degree of genome-wide mismatching of donor anti-host recognition between HLA-matched sibling transplants and HLA-matched unrelated donor transplants ^33^. On average, 17.3% of unrelated transplants were mismatched for coding SNPs compared to 9.4% of HLA-identical sibling pairs. HLA-matched unrelated donor transplants overall had low GVHD-related outcome risks. The risk was higher among HLA-DP mismatched unrelated donor transplants compared to HLA-matched sibling transplants. These data suggest that GVHD risk after unrelated donor transplantation is conferred in large part by HLA disparity through direct recognition of the mismatched HLA as well as HLA-peptide complexes. Future analysis of larger cohorts will be required to fully examine whether coding SNP variation is a robust proxy for overall degree of minor antigen mismatching and whether genome-wide patient SNP mismatching can contribute to GVHD after unrelated donor transplantation.

## Future considerations

The field of HLA continues to push the boundaries of transplantation genetics, from the perspective of basic understanding of the nature and organization of human genetic variation to genes that participate in the immune response. A greater appreciation for the biological implications of non-coding region variation will continue to provide insight into the relationship between the structure and function of MHC resident genes.
